# Vasculo-Neuronal Coupling and Neurovascular Coupling at the Neurovascular Unit: Impact of Hypertension

**DOI:** 10.3389/fphys.2020.584135

**Published:** 2020-09-25

**Authors:** Jessica L. Presa, Flavia Saravia, Zsolt Bagi, Jessica A. Filosa

**Affiliations:** ^1^Department of Physiology, Medical College of Georgia, Augusta University, Augusta, GA, United States; ^2^Departamento de Química Biológica, Facultad de Ciencias Exactas y Naturales, Universidad de Buenos Aires and Instituto de Biología y Medicina Experimental, CONICET, Buenos Aires, Argentina

**Keywords:** neurovascular, astrocyte, cogntive decline, ischemia, pulsatility

## Abstract

Components of the neurovascular unit (NVU) establish dynamic crosstalk that regulates cerebral blood flow and maintain brain homeostasis. Here, we describe accumulating evidence for cellular elements of the NVU contributing to critical physiological processes such as cerebral autoregulation, neurovascular coupling, and vasculo-neuronal coupling. We discuss how alterations in the cellular mechanisms governing NVU homeostasis can lead to pathological changes in which vascular endothelial and smooth muscle cell, pericyte and astrocyte function may play a key role. Because hypertension is a modifiable risk factor for stroke and accelerated cognitive decline in aging, we focus on hypertension-associated changes on cerebral arteriole function and structure, and the molecular mechanisms through which these may contribute to cognitive decline. We gather recent emerging evidence concerning cognitive loss in hypertension and the link with vascular dementia and Alzheimer’s disease. Collectively, we summarize how vascular dysfunction, chronic hypoperfusion, oxidative stress, and inflammatory processes can uncouple communication at the NVU impairing cerebral perfusion and contributing to neurodegeneration.

## The Neurovascular Unit: A General Overview

Blood flow in the brain is directed through a vascular network that links interconnected surface (pial) arteries to penetrating (parenchymal) arterioles and a vast network of capillaries, ultimately draining back to the surface via post-capillary venules and veins. Perfusion within the brain is a highly controlled process maintained through the operation of activity-dependent as well as constitutive mechanisms. Importantly, these mechanisms act in concert to ensure brain homeostasis and optimal functioning.

The brain is a complex functional and structural organizational unit. The smallest level of cellular interactions establishing brain perfusion is the neurovascular unit (NVU) (Iadecola, 2017), which comprises, at minimum, neurons, astrocytes, endothelial cells, vascular smooth muscle cells (VSMCs) and/or pericytes. Dynamic communication among these components contributes to the moment to moment regulation of arteriole and capillary blood flow and provides neurons with energy substrates as well as removal of waste metabolites. In the classic view, neuron-to-vessel communication (including via astrocytes), referred to as neurovascular coupling (NVC) and neurometabolic coupling, links increases in neuronal metabolic activity to increases in local blood flow (functional hyperemia). Importantly, interactions at the NVU are also bidirectional, with ongoing vessel-to-neuronal signaling, or vasculo-neuronal coupling (VNC), playing a role in establishing homeostasis at the NVU. In this review, we discuss dynamic signaling pathways at the NVU and how diseases, such as hypertension, affect them leading to cognitive dysfunction and neurodegeneration.

### Endothelial Cells

While the skull protects the brain from the external environment, endothelial cells are the first line of defense against the entrance of blood-borne pathogens, inflammatory-inducing components of the blood and circulating macrophages and are thus important contributors to brain homeostasis. The brain endothelium expresses highly specialized proteins, termed adherens and tight junction proteins, that mediate tight cell-to-cell contacts. These adherens (e.g., cadherins and junctional adhesion molecules) and tight junctions (e.g., occludins and claudins) contribute to formation of the blood–brain barrier (BBB), which maintains the high transendothelial electrical resistance and low paracellular transport properties characteristic of the brain endothelium (Sweeney et al., 2019; De Silva and Faraci, 2020). The basement membrane, comprising a family of proteins called perlecans (Thomsen et al., 2017), surrounds the endothelium and is also involved in homeostasis and maintenance of the BBB as well as communication at the NVU. Proteins of the basement membrane are known targets in disease; when their function is impaired, the integrity of the BBB is disrupted and the passage of molecules is dysregulated (Roberts et al., 2012; Mortensen et al., 2019). Mural cells and astrocytic endfeet surrounding the endothelium further contribute to maintenance of the BBB. In addition, endothelial cells release vasoactive signals, such as nitric oxide (NO), that control vascular resistance and thereby blood flow (Iadecola, 2013). The cellular mechanisms that drive endothelial-mediated changes in vascular tone include mechanical forces (e.g., wall shear stress), signals released by neighboring cells and electrical coupling such as endothelium-vascular smooth muscle cell (VSMC) interactions that drive hyperpolarization of the membrane potential of VSMCs, resulting in dilation (Longden et al., 2017). For example, agonist-induced activation of endothelial cell TRPV4 channels [i.e., epoxyeicosatrienoic acids (EETs)] increase calcium leading to the activation of intermediate (IK) and small (SK) conductance K^+^ channels which through myoendothelial junction causes hyperpolarization of VSMCs membrane potential resulting in vasodilation (Sonkusare et al., 2012). In addition, endothelial cells are electrically coupled via gap junctions allowing for the fast conductance of current along the endothelial syncytium, which at the arteriole level can also influence VSMC membrane potential (Figueroa and Duling, 2009; Bagher and Segal, 2011). A critical example of this process, discussed later in the review, is the activation of inwardly rectifying K^+^ (Kir2.1) channels in endothelial cells resulting in the upstream propagation of a hyperpolarizing current and dilation of parenchymal arterioles (Longden et al., 2017). For a detailed review of these mechanisms (see Harraz et al., 2020).

### Vascular Smooth Muscle Cells

As blood moves from larger arterioles downstream toward capillaries and then venules, its flow is regulated by crosstalk among various cells. Zonation—a gradual phenotypic change along the arteriovenous axis—was recently reported based on single-cell transcriptomics studies (Vanlandewijck et al., 2018). When applied to target disease-mediated changes in gene expression, transcriptomic studies such as this may help identify key drivers of small vessel disease and impaired communication at the NVU. In penetrating arterioles, a single layer of VSMCs wraps around the basal lamina, which in turn covers the entire endothelial cell layer. VSMCs play an essential role in the regulation of vascular tone (Hill-Eubanks et al., 2014) and also contribute to arterial elasticity by secreting extracellular matrix proteins (Wight, 2008).

### Pericytes

Pericytes are a pluripotent, perivascular mural cell type that is mainly found covering capillaries. Pericytes are also known to contribute to the maintenance and integrity of the BBB (Sweeney et al., 2019). They have further been proposed to regulate capillary blood flow by virtue of their contractile properties (Hall et al., 2014; Grutzendler and Nedergaard, 2019), a concept that is currently a matter of controversy largely owing to the lack of a consensus of phenotypic definition of SMCs versus pericytes. Functionally, it has been suggested that, during functional hyperemia, pericytes respond to glutamate released signals and dilate capillaries via a prostaglandin E_2_-dependent pathway (Hall et al., 2014). In support of a role for impaired neurovascular-mediated signaling, pericyte position and function are altered by disease conditions such as stroke (Hall et al., 2014; Berthiaume et al., 2018a,b), diabetes (Rom et al., 2020) and Alzheimer’s disease (AD) (Sagare et al., 2013; Nortley et al., 2019; Montagne et al., 2020), as well as disturbances in the circadian clock system (Nakazato et al., 2017)—all of which are associated with neurodegeneration.

### Astrocytes

Astrocytic endfoot processes make contact with the abluminal surface of mural cells, establishing the gliovascular interface (Simard et al., 2003). Astrocytes regulate cerebral blood flow (CBF) and contribute to the maintenance and integrity of the BBB. The multiple functions of astrocytes are reflected in the plethora of ion channels and receptors they express, as well as their active release of numerous vasoactive signals and gliotransmitters known to modulate vascular, microglial, and neuronal function (Araque et al., 2014). However, the nature of communication channels established between astrocytes, blood vessels, and neurons are unclear. The use of fluorescent dyes (Tsien, 1988, 1989) has enabled glioscientists to visualize astrocytic Ca^2+^ events (Cornell-Bell et al., 1990), giving rise to an upsurge in research and knowledge on astrocyte function and intercellular communication. Visualization of astrocyte Ca^2+^ dynamics was later improved with the development of Ca^2+^ indicators of the GCaMP series, either genetically encoded in transgenic mice or delivered via viruses (Bazargani and Attwell, 2016). The combined use of this approach with various astrocyte promoters such as Aldh1l1, Slc 1a3, GFAP, and GLAST has provided further knowledge on astrocyte heterogeneity in function, brain region specificity and structural dynamics.

Astrocytic Ca^2+^ events can be spontaneous or activity-dependent. As is the case for a number of astrocyte functions, Ca^2+^ events are *compartmentalized* (Bazargani and Attwell, 2016), with large Ca^2+^ events occurring in the soma and smaller events occurring in processes and microdomains. Importantly, these events can have different activity patterns. How these patterns relate to function is currently under investigation, supported by emerging sophisticated quantitative analytical techniques (Wang et al., 2019a). Dynamic Ca^2+^ events take the form of both intracellular- and intercellular-mediated signals, the latter of which can spread in the form of *waves* throughout the astrocytic syncytium via gap junctions (Munoz et al., 2015; Lapato and Tiwari-Woodruff, 2018). Ca^2+^ events may originate within different subcompartments, including the endoplasmic reticulum and mitochondria, and discrete sites, such as inositol trisphosphate (IP_3_) receptors. In addition, Ca^2+^ events may be initiated by activation of membrane-bound ion channels, receptors or transporters, such as vanilloid-type transient receptor potential channels (e.g., TRPV4) and Na^+^/Ca^2+^ exchangers. Notably, some of these surface proteins are functionally coupled to glutamate and GABA co-transporters (e.g., Glu/Na^+^, GABA/Na^+^ cotransporters), and thereby play an essential role in regulating ambient glutamate and GABA levels (for a review, see Oheim et al., 2018; Verkhratsky et al., 2019).

Astrocyte-derived vasoactive signals include prostaglandins, epoxyeicosatrienoic acids (EETs), glutamate, K^+^, adenosine and ATP, among others (Girouard and Iadecola, 2006; Attwell et al., 2010). The mechanisms by which each of these signaling molecules contribute to the regulation of vascular tone appear to be vessel specific (i.e., arteriole vs. capillary) (Mishra et al., 2016).

While the participation of astrocytic Ca^2+^ events in NVC has been questioned, mainly due to inconsistencies in the temporal relationship between Ca^2+^ events and vascular responses (Nizar et al., 2013; Bonder and McCarthy, 2014), these discrepancies could reflect unintended consequences of specific experimental manipulations, such as targeting a single Ca^2+^ pathway (IP_3_ receptor 2-knockout mice), failure to consider alternative Ca^2+^ sources [e.g., TRPV4 channels (Dunn et al., 2013)], anesthesia (Thrane et al., 2012; Tran and Gordon, 2015) and the arousal state of the animal (Tran et al., 2018). Technical limitations, such as the temporal and/or spatial resolution of acquired images, could also account for differences between studies. To this end, GCaMP-Ca^2+^ indicators have shed new light on this issue (Bazargani and Attwell, 2016). Using 3D imaging, Bindocci et al. (2017) discovered that Ca^2+^ events occurred more rapidly in astrocytic endfeet than in the soma. Global Ca^2+^ events associated with mouse movement were multifocal and spread from multiple gliapil toward the core of the cell. Endfoot Ca^2+^ events at the gliovascular interface exhibited asynchronous patterns (Bindocci et al., 2017). Moreover, astrocytic Ca^2+^ transients have been shown to propagate between cell subcompartments, highlighting the diversity of responses (Grosche et al., 1999; Kanemaru et al., 2014). In an awake *in vivo* two-photon mouse model, Tran et al. (2018) evaluated astrocyte Ca^2+^ dynamics in peri-arterioles and peri-capillary astrocytes of the barrel cortex in response to sensory stimulation or volitional behaviors. The authors observed delayed (following dilations) astrocyte Ca^2+^ increases in turn dependent on the mouse’s action at the time of the stimulus. Using varied stimulation approaches the authors showed that the neurovascular pathway was linked to endothelial-derived nitric oxide (Tran et al., 2018). Unraveling the functional significance of astrocytic Ca^2+^ activity patterns—and sources (Srinivasan et al., 2015)—under both physiological and non-physiological conditions is thus of critical importance. In addition, further studies designed to link activity patterns with astrocyte population subtype are needed (Sofroniew and Vinters, 2010; Lundgaard et al., 2014).

Astrocytes are also critically involved in removing waste from the brain via the glymphatic system, a process that involves the highly specific endfoot marker and water ion channel, aquaporin 4 (AQP4). The glymphatic system operates through vessel-to-glia crosstalk mediated, at least in part, by arteriole pulsatility and impaired in aged mice (Kress et al., 2014). Although this latter mechanism is not fully established, it has been proposed that these pulsations are the driving force for promoting the convection of ions, solutes and waste products across the brain interstitial space without the need to cross the BBB. There is some evidence that the absence of AQP4 at the gliovascular interface impairs clearance and transport via the glymphatic system (Iliff et al., 2012, 2013).

### Microglia

Microglia are the resident immune cells of the brain (Bilbo and Stevens, 2017). These embryonic mesoderm-derived cells constantly surveil the CNS for unfamiliar antigens, pathogens, apoptotic cells, and foreign particles. Their dynamic phagocytic activity and active release of immune signals play critical roles in brain homeostasis and repair processes (Hanisch and Kettenmann, 2007). However, the neuroprotective microglial state (surveillance/execution mode) can be disturbed during pathology, causing these cells to shift toward a reactive phenotype. These reactive microglia respond with exacerbated secretion of proinflammatory mediators (Vinuesa et al., 2019) and altered phagocytotic properties (Baik et al., 2019; Zhang et al., 2020).

Whether microglia directly impact cerebrovascular function is poorly understood. It has been reported that, after a cortical stroke, microglia are recruited toward vessels, where they can phagocytize endothelial cells and thereby contribute to the increased loss of BBB integrity (Jolivel et al., 2015). Using an *in vitro* approach, the authors of this latter study provided evidence that fibrinogen and albumin, plasma components found in the brain parenchyma after stroke, exacerbated the secretion of microglial proinflammatory cytokines and acted as chemoattractants. However, whether phagocytosis of endothelial cells by microglia, a potential step in vascular repair, is part of a normal physiological process remains unknown.

To investigate this, Halder and Milner (2019) used a mouse model of mild chronic hypoxia that causes vascular leakiness and microglia clustering around vessels. Under these hypoxic conditions, pharmacological depletion of microglia increased BBB leakiness and caused a loss of tight junction proteins. It also led to astrocyte-vessel uncoupling, as evidenced by a decrease in the number of vessels positive for the perivascular astrocyte endfoot marker, AQP4. However, under normoxic conditions, microglia depletion did not impact vascular integrity. Thus, it is likely that coupling of these interactions is dependent on key signals released only in a pathological setting that may be absent under physiological conditions.

As alluded to above, proper operation of homeostatic mechanisms involves dynamic crosstalk among cells of the NVU, many of which have overlapping or complementary functions. These observations call for an integrated approach for addressing the functional status of the NVU and its vulnerability under disease conditions.

## Cerebral Blood Flow, Neurovascular Coupling, and Vasculo-Neuronal Coupling

High energy demands, as occur during information processing and maintenance of homeostatic processes, are sustained by redundant NVU-mediated mechanisms that ensure constitutive perfusion (Zlokovic, 2005). The driving force for CBF is cerebral perfusion pressure (CPP), defined as the difference between mean arterial pressure (MAP) and intracranial pressure (ICP). In the absence of a neuronal stimulus, CBF is relatively constant, but can be affected by changes in cardiac output, vascular resistance and altered intravascular as well as intracranial pressure gradients.

### Cerebral Autoregulation

The dynamic mechanisms that regulate basal CBF are largely driven by the fine balance between vasodilatory and vasoconstrictive signaling pathways (Hill-Eubanks et al., 2014) and the integration of reflex autonomic pathways and local control processes, including cerebral autoregulation (Bayliss, 1902; Silverman and Petersen, 2020). The mechanisms underlying cerebral autoregulation include pressure-induced vasoconstriction (myogenic response) (Brayden et al., 2013; Cipolla et al., 2014; Pires et al., 2015) as well as metabolic and humoral signaling (Koller and Toth, 2012). The best-studied of these cellular pathways is the myogenic response, which involves activation of mechanosensitive Ca^2+^-permeable ion channels and a subsequent increase in Ca^2+^ influx into VSMCs, resulting in elevated intracellular Ca^2+^ and vasoconstriction (Hill et al., 2006; Cipolla, 2009; Li et al., 2014). In cerebral vessels, this process is both pressure and flow/wall shear stress dependent (Koller and Toth, 2012).

Under physiological conditions, these dynamic mechanisms operate within a MAP range of 50 to 160 mmHg and serve to adjust the diameter of arterioles to maintain approximately constant CBF (Silverman and Petersen, 2020). However, the actual systemic arterial pressure range corresponding to this plateau phase varies (Faraco and Iadecola, 2013), with potentially significant differences reported between individuals (Tzeng et al., 2010; Liu et al., 2016; de Jong et al., 2017; Xing et al., 2017; Elting et al., 2020). Moreover, chronic diseases, such as hypertension and cerebral ischemia, can shift this autoregulatory range. Consequently, impairment of autoregulatory mechanisms that buffer blood pressure fluctuations can increase the vulnerability of the microcirculation, and hence the NVU (Iadecola, 2017). Cerebral autoregulation can be assessed by measuring changes in CBF in response to physiological stimuli (e.g., exercise, hypercapnia or orthostatic challenges), a response referred to as cerebrovascular reactivity (Lavi et al., 2006). Cerebrovascular reactivity is reduced in hypertension and large-artery stiffness is increased (Chirinos et al., 2019; Marmarelis et al., 2020; Silverman and Petersen, 2020), both of which contribute to cognitive decline, as we discuss below in more detail in this review article.

### Neurovascular Coupling

In contrast to cerebral autoregulation, which constitutively regulates CBF, NVC is a dynamic process characterized by robust vascular responses to vasoactive signals released upon local increases in neuronal activity. NVC mechanisms form the basis of functional hyperemic responses. The rapid redirection of blood flow driven by increases in neuronal activity ensures the targeted, uninterrupted flow of blood that is critical for brain viability and ongoing brain functions. Details of the proposed signals underlying NVC have been extensively reviewed previously (Iadecola, 2017; Hosford and Gourine, 2019).

While the sequence of events underlying communication among the various cells of the NVU is poorly understood, the current view is that capillaries and arterioles are critical for the *initiation* (Mishra et al., 2016; Rungta et al., 2018) and *conduction/integration* (Longden et al., 2017) of NVC responses. Longden et al. (2017) recently established an essential role for capillary endothelial cells in both initiation and conduction of NVC responses, showing that activation of Kir2.1 channels in capillary endothelial cells by extracellular K^+^, which is released during each action potential, generates a hyperpolarizing (electrical) signal that back-propagates to cause dilation of upstream penetrating arterioles and pial arteries, thereby inducing an increase in blood flow to the site of signal initiation (Tian et al., 2010; Chen et al., 2011, 2014; Longden et al., 2017; Cai et al., 2018; Rungta et al., 2018). Notably, this hyperpolarizing current travels millimeters before dissipating (Iadecola et al., 1997; Chen et al., 2011). This spatial spread of the vascular signal relative to the initiation point could lead to a mismatch between the site of elevated neural activity and the location of vascular responses, highlighting the potential role of additional mechanisms at different levels of the vascular network in decoding functional hemodynamics (Jukovskaya et al., 2011; O’Herron et al., 2016). Indeed, using two-photon imaging, O’Herron et al. (2016) demonstrated that neuronal activity was more strongly coupled to the responses of nearby parenchymal arterioles than with the responses of upstream pial vessels. Interestingly, it is conceivable that the propagating dilatory signal not only moves upstream to the surface but also travels from the surface back down into penetrating branches. Though this aspect of vascular signaling has received little attention, low-resolution vascular imaging suggests that it readily occurs (Chen et al., 2011), providing a possible explanation for the residual mismatch between neural activity and nearby vascular responses.

There is also evidence supporting pericytes and VSMCs as first responders (Hall et al., 2014; Mishra et al., 2016). Using brain slices, Mishra et al. (2016) showed that neuronal activity evokes increases in astrocytic Ca^2+^ (via activation of the ionotropic ATP receptor, P2X_1_) that stimulate the synthesis of prostaglandin E2 (PGE_2_) and its release from astrocytic endfeet, resulting in capillary dilation through activation of EP_4_ receptors on pericytes. However, in a follow-up study using two-photon *in vivo* imaging, Rungta et al. (2018) assessed changes in intracellular Ca^2+^ in pericytes and VSMCs in relation to distinct capillary and arteriole Ca^2+^ levels induced by olfactory neuronal activation (Rungta et al., 2018). In this study, an odor stimulus was followed by fast, synchronized decreases in Ca^2+^ in arterioles and first-order capillaries (less than ∼50 μm from the arteriole) (Rungta et al., 2018). It was determined that pericytes near the activated synaptic site responded with a delayed drop in Ca^2+^ as opposed to mural cells upstream. Thus, the authors proposed the drop in Ca^2+^ was likely generated from heterocellular electrical coupling between endothelial and mural cells via a retrograde hyperpolarization and transfer of current via myo-endothelial junctions (Iadecola et al., 1997; Chen et al., 2014; Longden et al., 2017; Harraz et al., 2020). Collectively, these studies support capillary endothelial cells as the initiators of the NVC response.

Another mechanism shown to contribute to the initiation of functional hyperemic responses involves changes in the velocity of red blood cells (Wei et al., 2016), a process that is independent of NVU vasoactive signals (Grutzendler and Nedergaard, 2019). Wei et al. (2016) provided evidence that the transient decrease in tissue O_2_ tension resulting from neuronal-evoked increases in metabolic activity (Devor et al., 2011) increases red blood cell deformability and velocity in capillaries. The authors proposed that O_2_ release-induced displacement of ankyrin from band 3 weakens spectrin-actin cytoskeleton interactions (Stefanovic et al., 2013; Zhou et al., 2019), thereby increasing erythrocytes deformability (Wei et al., 2016), a phenomenon later confirmed using both *ex vivo* (microfluidics) and *in vivo* (two-photon imaging) approaches (Zhou et al., 2019).

Understanding the cellular mechanisms underlying efficient NVC is crucial for the development of therapeutic approaches targeting potential mismatches in metabolic demand and supply as occurs in disease conditions, making this an active area of investigation.

### Vasculo-Neuronal Coupling

While NVC mechanisms act to communicate information from neurons to the vasculature, communication in the opposite direction has also been proposed. At the cellular level, this modality of communication is referred to as VNC. This concept, encapsulated in the hemo-neural hypothesis, posits that hemodynamic signals modify glial and neuronal function to maintain cerebral homeostasis and contribute to information processing in the brain (Moore and Cao, 2008; Cao et al., 2009). In support of vascular-to-neuron signaling in the brain, Waki et al. (2006) showed that inhibition of eNOS in the nucleus tractus solitarii (NTS) decreased arterial pressure in the spontaneously hypertensive rat (SHR) model, indicating a role for endothelial-derived NO in the NTS in modulating cardiac baroreceptor reflex gain and arterial pressure. Using acute rat brain slices and a mechanical stimulation paradigm that targets endothelial cells, Kozlov further demonstrated that VSMCs and astrocyte endfeet generate inhibitory, slow, outward currents in mitral cells of the olfactory bulb that are mediated by GABA released from astrocytes (Kozlov et al., 2006). Thesen et al. (2012) provided indirect experimental evidence in favor of the hemo-neuronal hypothesis in humans, demonstrating changes in neuronal activity in response to hypercapnia (5% CO_2_). Specifically, they showed that hypercapnia, a prominent inducer of vascular dilation, decreased the magnetoencephalogram response to auditory and visual tasks (Thesen et al., 2012), reducing both evoked and spontaneous spectral power indicative of neuronal activity. These data support a link between resting perfusion status and neuronal activity and highlight the importance of considering this linkage in assessments of activity-dependent changes in blood flow. However, because pH acidification can independently affect blood vessels and neuronal activity, the concept will require further refinement.

Using a brain slice model, our group showed that arteriole constriction is associated with inhibition of neuronal firing, whereas dilation is linked to activation of neuronal firing. A detailed investigation of the constriction-dependent pathway revealed the involvement of TRPV4 channel-mediated, Ca^2+^-dependent events in astrocytes (Kim et al., 2015, 2016). While the mechanisms are incompletely understood, the constriction-evoked inhibition in neuronal activity was unaffected by blockers of NO, GABA_*A*_ receptors or glutamate receptors. On the other hand, the response was significantly blunted by an adenosine A1 receptor blocker, and to some extent by blockade of GABA_*B*_ receptors (Kim et al., 2016).

Both arteriole constriction and dilation induce changes in astrocytic Ca^2+^, independent of neuronal activity (Kim et al., 2015; Rosenegger et al., 2015). One possible mechanism for this is that changes in vessel diameter trigger activation of mechanosensitive ion channels, or receptors, at the NVU. The resulting increase in astrocytic Ca^2+^ could then engage downstream pathways, leading to changes in neuronal activity. While evidence supports mechanosensitivity in astrocytes, how this might influence neurovascular interactions is unclear, as these processes remain largely unexplored. Mechanostimulus-induced changes in astrocytic Ca^2+^ could be attributable to a number of different ion channels, including P2 × 7 (Beckel et al., 2014; Albalawi et al., 2017), TRPV4 (Ryskamp et al., 2011; Dunn et al., 2013; Choi et al., 2015), Piezo1 (Choi et al., 2015; Velasco-Estevez et al., 2020), and pannexin1 (Minelli et al., 2007; Suadicani et al., 2012). Evidence also supports the idea that mechanical stimulation causes the release of adenosine in the brain. Further studies addressing the implications of mechanical distortion-driven signaling at the NVU are clearly warranted. A good starting point would be the gliovascular interface, where specialized ion channels reside and where arteriole pulsation may play an important role in conveying mechanoregulation-driven communication (Ross et al., 2014; Nguyen and Venton, 2015; Wang and Venton, 2019; Wei et al., 2019).

We proposed VNC as a constitutively active mechanism involved in the modulation of resting neuronal activity that contributes to matching perfusion levels with energy demand. In this view, VNC would serve to safeguard neurons in cases where perfusion was compromised. A further prediction is that, in the healthy brain, VNC is overridden during increases in neuronal activity, such as those evoked during NVC. If VNC is linked to mechanosensitivity at the NVU, its operation could be further affected by intracranial and intravascular pressure gradients, which have recognized relevance in stroke and traumatic brain injury. The potential intersection of such pressure gradients with VNC could also be important in the context of hypertension, vascular stiffness and small vessel disease, an issue that warrants further investigation.

## Vascular Risk Factors and the NVU

### Hypertension

Genetic, epigenetic, and lifestyle factors contribute to hypertension (Padmanabhan et al., 2015). Also, the prevalence and severity of hypertension increase with age. From age 20–85 years of age, the lifetime risk of hypertension has been estimated at 69.3–86.1% depending on sex and race (Virani et al., 2020). With the world’s older population rising, the number of older adults with hypertension is expected to grow by 20% by 2050 (Benetos et al., 2019; Volpe et al., 2019). Yet, many older adults remain untreated or the management of their hypertension challenging to address. In the brain, chronically increased arterial pressure leads to lacunar infarcts, white matter hyperintensities, and cerebral microbleeds (Joutel and Faraci, 2014). Hypertension also results in blood vessel remodeling, increased cerebrovascular resistance, cerebral hypoperfusion and impaired NVC (Girouard and Iadecola, 2006; Calcinaghi et al., 2013; Alosco et al., 2014; Hu et al., 2017), making it a risk factor for small vessel disease (Joutel and Chabriat, 2017) and a leading contributor to stroke. Importantly, untreated mid-life hypertension is a modifiable risk factor for the early development of dementia (Mogi, 2019).

In response to elevated intravascular pressure, arteries undergo various remodeling processes, including hypertrophic remodeling, hypotrophic remodeling, inward remodeling, and arterial stiffening (Baumbach and Ghoneim, 1993; Intengan and Schiffrin, 2001; Pires et al., 2013). The most-studied cerebral vascular beds in hypertension include branches of the internal carotids and vertebral arteries, which perfuse the brain and cerebellum/brainstem, respectively. Hypertension differentially affects these two branches of the circulation, with vertebral artery remodeling preceding that of the common carotids (Dickinson and Thomason, 1959). These anatomical and functional observations support the idea that brain areas that are home to cardiovascular-related control centers may be the first targets of the disease. Thus, hypertension leads to regional variations in CBF (i.e., hypoperfusion) and, consequently, changes in neuronal output function.

Importantly, hypertension also alters the normal function of parenchymal arterioles—the ultimate effector of parenchymal perfusion (Iddings et al., 2015; Pires et al., 2015; Sweet et al., 2015; Diaz et al., 2019). Parenchymal arterioles from SHRs and their stroke-prone SHRSP derivatives exhibit increased tone (Iddings et al., 2015; Pires et al., 2015; Sweet et al., 2015), increased wall thickness and passive distensibility (Sweet et al., 2015), decreased resting lumen diameter, and inward remodeling (Pires et al., 2015). Our group also reported increases in parenchymal arteriole tone in *ex vivo* brain slice preparations from mice infused with Ang II (600 ng/kg/min) for 28 days (Diaz et al., 2019). In addition, DOCA-salt–induced hypertension has been shown to impair endothelium-dependent dilation via dysfunctional cyclooxygenase (COX) and NO signaling (Matin et al., 2016).

It has previously been shown that the reduced cerebral perfusion and pathological parenchymal arteriole remodeling are reversed by endothelial cell-specific knockout of the mineralocorticoid receptor (MR) or MR antagonism in mice (Diaz-Otero et al., 2017). Diaz-Otero and colleagues showed that MR activation impairs TRPV4 channel-mediated dilation of parenchymal arterioles in a mouse model of angiotensin II (Ang II)-induced hypertension. The authors also reported that, in the hypertensive group, MR blockade rescued myogenic tone, reduced microglia density, and improved cognitive function (Diaz-Otero et al., 2018). The authors then evaluated the role of TRPV4 channels in vessel dysfunction using a TRPV4-knockout rat model [WKY-Trpv4(em4Mcwi)], reporting reduced cerebral perfusion with no changes in parenchymal arteriole structure or myogenic tone. However, these rats did show impaired endothelium-dependent dilation and cognitive function in association with gliosis (Diaz-Otero et al., 2019). It has further been shown that relaxation of VSMCs is diminished in mesenteric arteries of SHR and SHRSP models; this effect was attributed to a decrease in the generation of the endothelium-dependent hyperpolarizing factor, a process that likely depends on TRPV4 and SK channel signaling (Seki et al., 2017). TRPV4 channel activity was further evaluated by Tajada et al. (2017) using super-resolution imaging. These authors showed that TRPV4 channel activity was dependent on the protein kinase Cα (PKCα) membrane-anchoring protein AKP150, and further demonstrated that Ang II produced a decay in TRPV4 activation that was negatively correlated with the distance between TRPV4 channels and AKAP.

Yamasaki et al. (2020) showed that angiotensin II type 1 receptors (AT_1_Rs) were differentially expressed between pial arteries and parenchymal arterioles of the cerebral microvasculature. These authors reported that, whereas mRNA expression levels of the angiotensin II receptor subtypes AT_1_R_*a*_ and AT_1_R_*b*_ were comparable in parenchymal arterioles, expression levels of AT_1_R_*b*_ were ∼11-fold higher than those of AT_1_R_*a*_ in pial arteries. These differences in expression suggest differences in the sensitivity of these vessels to Ang II (Yamasaki et al., 2020). However, how expression of these receptors is altered throughout the different stages of hypertension was not evaluated in this study. An important functional observation was that AT_1_R activation in parenchymal arterioles is important for the initiation, but not the maintenance, of the myogenic response. The authors suggested that a G protein-coupled receptor (GPCRs), such as AT_1_R, acts as the main intravascular pressure sensor, providing a molecular pathway critical for cerebral autoregulation (Yamasaki et al., 2020).

Hypertension also targets cerebral capillaries. Hypertension impairs endothelial function and BBB integrity (Santisteban and Iadecola, 2018). Both animal models of hypertension (Tarantini et al., 2016) and hypertensive patients (Bosch et al., 2017) show capillary rarefaction. Loss of capillaries is associated with ischemia resulting in inadequate metabolic supply to neurons and glial cells contributing to elevated oxidative stress and inflammation and an overall toxic environment. There’s also a synergistic effect of hypertension with both aging and AD on microvascular damage (Toth et al., 2013; Tarantini et al., 2016). Accompanied by a decrease in the number of upstream arterioles (De Silva and Faraci, 2020), hypertension contributes to an overall reduction in cerebral perfusion. Because endothelial dysfunction is a hallmark of hypertension, it would be important to address whether hypertension alters the electrical conduction of endothelial cells and, thus, the ability of capillaries to initiate the propagation of the upstream vasodilation, characteristic of the NVC response (Chen et al., 2014; Longden et al., 2017). A critical factor associated with the retrograde upstream propagation of the hyperpolarizing current is phosphatidylinositol 4,5-bisphosphate (PIP_2_). Gq-protein-coupled receptors, targets of numerous NVC mediators, evokes PLC-mediated hydrolysis of PIP_2_ into IP_3_ and DAG, which in turn modulate ion channels involved in the regulation of vascular tone (Harraz et al., 2018b) including Kir2.1 and TRPV4 ion channels (Harraz et al., 2018a,b; Moshkforoush et al., 2020). Given that ischemia alters oxidative phosphorylation and ATP synthesis, the impact of PIP_2_-mediated ion channel regulation on the vasculature is of importance (Harraz et al., 2018b). Future work on this research area will help elucidate the effect of aging, as well as hypertension-induced capillary dysfunction, has on the mechanisms underlying neurovascular uncoupling.

Collectively, these studies support the concept that parenchymal arterioles and capillaries are a target of hypertension, which manifests as increased tone and endothelial dysfunction, and is accompanied with gliosis (Iddings et al., 2015; Pires et al., 2015; Sweet et al., 2015; Diaz et al., 2019).

### Hypoperfusion and Oxidative Stress in Hypertension

Reduced CBF is correlated with accelerated cognitive decline and ultimately leads to dementia (Hakim, 2019). Using MRI analyses and clinical history data for 59 patients who suffered a transient ischemic attack, Wang and colleagues reported that 67.8% of such patients presented a history of hypertension. They also observed a strong correlation between hypertension and perfusion abnormalities that was independent of the ischemic attack (Wang et al., 2019b). Hypertension, together with hypoperfusion, can result in significant dysregulation of cerebral vessels, increasing vascular resistance and exacerbating myogenic constriction (Iddings et al., 2015). As part of a consequent, and presumably protective, response mechanism, hypertension shifts the cerebral autoregulation curve to the right (Koller and Toth, 2012), an adaptation that also increases the risk for hypoperfusion when systemic blood pressure decreases (Faraco and Iadecola, 2013; Toth et al., 2017). However, these protective adaptations may be lost in the aged brain. Using an Ang II-infused model of hypertension in mice, Toth et al. (2013) investigated the effects of increased pressure-induced tone in middle cerebral arteries of 3-month-old mice. They found that the mechanism governing increased tone involved upregulation of the vasoconstrictor pathway driven by 20-HETE (20-hydroxy-5,8,11,14-eicosatetraenoic acid) and the canonical transient receptor potential cation channel member, TRPC6. However, in aged mice (24 months), this mechanism was impaired, increasing the vulnerability of the cerebral microcirculation to blood pressure fluctuations (Toth et al., 2013).

Chan et al. (2019) recently provided support for a role for endothelial TRPV4 channels in the regulation of parenchymal arteriole tone and vascular remodeling during hypoperfusion. In these studies, wild-type and TRPV4-knockout (KO) mice were subjected to unilateral common carotid artery occlusion (UCCAo) for 28 days. In wild-type mice, UCCAo caused a decrease in active tone and increased passive inner diameters (Chan et al., 2019). The decreased tone was dependent on TRPV4 channel function but not the outward remodeling. These effects of UCCAo on myogenic tone did not involve NO signaling. The same group showed decreased myogenic tone in control WKY rats subjected to UCCAo, an effect that was not observed in SHRSP model animals (Sweet et al., 2015). Together, these data suggest that ischemia, as well as hypertension, result in the activation of distinct compensatory processes. While ischemia-related signaling is geared toward maintaining vessels open and sustaining CBF, hypertension-induced increases in vascular tone serve to prevent hyperperfusion-induced microvascular damage. In hypertension, remodeling and increased vascular reactivity may lead to hypoperfusion and the onset of cerebral ischemia.

Reactive oxygen species (ROS), which are elevated in hypertension (Montezano et al., 2015), are involved in vascular inflammation, fibrosis and arterial remodeling (Tanaka and Laurindo, 2017), and thus contribute to vascular dysfunction (Lopes et al., 2015). Using a transcriptomic approach, Walas et al. (2019) presented evidence that, prior to the onset of hypertension, cerebral arteries from the posterior circulation of 5-week-old SHRs exhibit elevated expression of genes related to remodeling, oxidative stress, and inflammatory pathways, notably including a 40% increase in genes related to fibrosis (e.g., TGFβ). Among the changes noted during this pre-hypertension period were dysregulation of pro-fibrotic pathways (e.g., upregulation of enolase-1) and inflammatory pathways (e.g., inhibition of interferons, TLR3 and STAT1) as well as altered mitochondrial function. The authors proposed that these changes support previously reported immune cell infiltration (Waki et al., 2007, 2013; Xu et al., 2012; Marvar et al., 2016). The resulting vessel remodeling alters vascular function, increasing cerebrovascular resistance and decreasing CBF, both of which contribute to increased sympathetic nerve activity via the Cushing mechanism, as recently reviewed (McBryde et al., 2017). The onset of hypertension was thus regarded as a secondary insult that worsened vessel fibrosis and stiffening and also caused endothelial dysfunction. These results obtained using the SHR model suggest that early therapeutic approaches targeting immune system dysfunction may prevent vascular dysfunction associated with hypertension.

Increased oxidative stress and resulting endothelial dysfunction are also associated with decreased CBF and increased BBB permeability (Iadecola, 2013). Moreover, hypertension is correlated with the size and prevalence of microbleeds (Vernooij et al., 2008; Shams et al., 2015)—a marker of vessel damage—and BBB breakdown. Increased ROS levels promote increases in intracellular astrocytic Ca^2+^, which has been linked to the ability of astrocytes to sense hypoxia (Angelova and Abramov, 2016). In the context of exacerbated ROS levels in disease, this mechanism may contribute to the pathogenesis of neurodegenerative disorders.

In mice, Ang II-dependent hypertension impaired NVC-evoked increases in CBF and endothelial-mediated vasodilation (Capone et al., 2012; Johnson et al., 2013). Ang II-induced hypertension is associated with increased activation of NADPH oxidase and enhanced ROS production (Kazama et al., 2004; Capone et al., 2011). Iulita et al. (2019) provided evidence that in a murine Ang II-dependent model of hypertension (1000 ng/kg/min), the adoptive transfer of Treg cells prevented neurovascular uncoupling and endothelial dysfunction. Neuroprotective effects were linked to IL-10 signaling and abolished in IL-10 deficient mice. Notably, the adoptive transfer of Tregs reduced Ang II-induced systemic inflammation, microgliosis and superoxide radicals (Iulita et al., 2019). Faraco et al. (2016), investigated the contribution of perivascular macrophages (PVMs), resident brain macrophages with the capacity to generate ROS, to hypertension-induced neurovascular and cognitive dysfunction. Low Ang II doses was shown to increase BBB permeability and Ang II access into perivascular spaces. The subsequent PVM activation, via AT1R and the NADPH oxidase subunit NOX2, contributed to ROS production (Faraco et al., 2016). The authors further demonstrated that in BPH/2J mice, PVM depletion improved cognitive impairments, providing an important link between hypertension, PVM/inflammation, and cognitive dysfunction (Faraco et al., 2016). The same group recently demonstrated that Ang II-induced hypertension in mice leads to tight junction remodeling and increased BBB permeability, an effect more pronounced in arterioles than capillaries (Santisteban et al., 2020). The authors proposed a mechanism by which circulating Ang II leads to AT1a receptor activation in endothelial cells and downregulation of *Mfsd2a.* The resulting increased vesicular transport of Ang II into the perivascular space would then lead to AT1R and NOX2 activation in PVM, increasing BBB disruption (Santisteban et al., 2020).

### Inflammation, Aberrant Astrocytic Ca^2+^ and Disease

While astrocytes are known for their trophic support, these cells also participate in proinflammatory processes (Pomilio et al., 2016; Verkhratsky and Nedergaard, 2018). Alteration in glial function and morphology (i.e., astrogliosis) is associated with disease states, vasodilator dysfunction of cerebral parenchymal arterioles (Bagi et al., 2018) and cognitive dysfunction (Verkhratsky and Nedergaard, 2018). Hypertension, regarded as a chronic inflammatory state (De Miguel et al., 2015; Rodriguez-Iturbe et al., 2017), impairs one of the first barriers of brain protection, the BBB (Katsi et al., 2020; Santisteban et al., 2020). BBB dysfunction facilitates the infiltration of plasma components to the brain and the production of proinflammatory signals that lead to microglia and astrocyte activation. These inflammatory processes impair the glial trophic support of both the vasculature and neurons (Iadecola, 2013). The extensive coverage of astrocytic processes along the cerebral microcirculation could be regarded as the next in line protective barrier (Sofroniew, 2015). However, how endothelial dysfunction alters the functional properties of astrocytes remains poorly understood. We speculate that the progressive impairment of the cerebral microcirculation, accompanied by the release of inflammatory markers, contributes to a shift in astrocyte phenotype from protective to inflammatory. Consequently, neuroinflammation directed by glial cells may contribute to hypertension-related cognitive impairment.

Efforts have been made to delineate astrocyte functional phenotypes based on the expression of specific markers, as previously reviewed (Miller, 2018). To this point, however, molecular profiling strategies (e.g., single-cell transcriptomics), have not been able to fully determine astrocyte functional status. Despite transcriptomic comparisons between astrocytes positive for GFAP, ALDH1L1, GLT1 or HEPACAM, the co-expression of markers in the majority of astrocytes has limited the ability to identify astrocyte subpopulations (Lovatt et al., 2007; Yang et al., 2011; Zhang et al., 2016).

To date, two major astrocyte phenotypes have been described based on their susceptibility to signals from microglia. A1 astrocytes, analogous to the controversial M1 microglia subtype, develop a proinflammatory phenotype that cannot support synapse formation or phagocytosis. The A1 phenotype is primarily driven by interleukin (IL)-1α, tumor necrosis factor (TNF)-α, and C1q (complement component 1, subcomponent q) (Liddelow et al., 2017). On the other hand, trophic or protective astrocytes, referred to as A2 astrocytes, represent the normally functioning astrocyte subtype. The A2 astrocyte phenotype is also linked to the promotion of neuronal survival following ischemia (Liddelow et al., 2017; Clarke et al., 2018). Importantly, whether astrocytes can reversibly switch from one phenotype to the other is presently unknown.

Given limited knowledge on the factors leading to astrocytes functional transition from supportive to proinflammatory, alternative approaches, including incorporation of an assessment of Ca^2+^ dynamics, are pivotal for interpreting astrocyte function and population subtype. Increasing evidence pointing to aberrant astrocytic Ca^2+^ as an early indicator of disease state and severity strengthens the importance of decoding astrocyte communication at the NVU for optimal brain function (Shigetomi et al., 2019).

Models of cerebrovascular pathology such as stroke (Morrison and Filosa, 2016; Morrison and Filosa, 2019) and AD, show increases in astrocyte Ca^2+^ events (Delekate et al., 2014). Less understood, however, is the impact of hypertension on astrocyte Ca^2+^ dynamics and function. Ohara and Nabika (2016) identified a point mutation in stromal interaction molecule-1 (Stim1) in the SHRSP model, and further showed that truncated STIM1 impaired Ca^2+^ signaling regulated by store-operated Ca^2+^-entry in cultured astrocytes. Diaz et al. (2019) showed augmented spontaneous and myogenic-evoked Ca^2+^ events in microdomains of cortical astrocytes from Ang II-induced hypertensive mice, events that were accompanied by increased TRPV4 channel function. Although it is not entirely clear how this contributes to pathogenesis, the authors hypothesized that sustained intracellular Ca^2+^ elevations in astrocytes could trigger or facilitate the transition of these cells to a proinflammatory state. In support of this, exacerbated TRPV4 channel activation has been linked to the release of proinflammatory signals (Krizaj et al., 2014) that may, in turn, initiate astrocyte-microglia interactions (e.g., ATP, DAMPs), establishing a feed-forward response that aggravates neuroinflammation at the NVU.

In AD, astrocytes in the vicinity of amyloid plaques exhibit abnormally elevated Ca^2+^ levels (Shigetomi et al., 2019). Astrocytes in acute brain slices also respond to exposure to Aβ oligomers with an increase in intracellular Ca^2+^ (Tyurikova et al., 2018). Similar findings have been reported in stroke, were astrocytes in proximity to damaged tissue show increased Ca^2+^ (Shigetomi et al., 2019). Notably, suppression of aberrant astrocytic Ca^2+^ signals in the context of stroke reduces cell and tissue damage (Ding et al., 2009), indicating that astrocyte Ca^2+^ overload can lead to pathology. Further studies are needed to uncover mechanistic details of altered astrocytic Ca^2+^ signaling in AD models.

### Vascular Stiffness

There are several human studies and observations indicating that hypertension and aging exert synergistic effects on cognitive decline (Csiszar et al., 2013). In fact, hypertension may prompt the early onset of mechanisms underlying accelerated aging. Both hypertension and aging increase vascular stiffness. In healthy elastic arteries (thoracic aorta and common carotid artery), the compliance of these vessels dampens pressure and flow fluctuations, thereby allowing delivery of a steady flow of blood to the more vulnerable microcirculation (Chirinos et al., 2019). Sustained mechanical stress causes arteries to lose their distensibility, leading to increased stiffness (Avolio et al., 2018; Mitchell, 2018). Thus, both age and hypertension result in a reduction in pressure-buffering capacity upstream from the cerebral microcirculation. As a consequence, the amplitude of pulsations transmitted to the carotids is increased (Mitchell et al., 2011). At the microcirculation level, hypertension contributes to small vessel disease (Faraci, 2018). Moreover, hypertension results in increase wall stiffness of larger vessels (i.e., posterior cerebral artery) and increased wall stress and wall thickness of parenchymal arteriole (Diaz-Otero et al., 2016). While few have addressed the importance of understanding the relationship between the macro- and micro-vasculature, these questions are emerging (Climie et al., 2019) and constitute new paradigms toward the development of new therapeutic targets addressing the impact of cardiovascular disease on cognitive function.

Arterial stiffness is a predictor of cardiovascular risk. In clinical practice, the gold standard for measurements of regional arterial stiffness is pulse wave velocity (PWV) (Negoita et al., 2018). The PWV, often measured from the carotid to the femoral artery, is defined as the distance traveled by the pulse wave from the carotid point to the femoral point (ΔL) divided by the time of travel (ΔT) or, PWV = ΔL/ΔT. In the aging population, increased pulse pressure is also linked to hypertension. To this end, measurements of wave intensity and wave separation analysis (Fok et al., 2014; Su et al., 2017) allow pressures to be separated into the push forward (from the ventricle against the arterial tree) and backward (from arterial tree against the ventricle) components (Fok et al., 2014). Thus, different components of the pressure wave can provide information related to the pressures generated from the ventricle and the arterial tree.

Arteries from hypertensive patients exhibit increased stiffness and a loss of distensibility, which increases their susceptibility to pulse pressure-mediated damage (Lacolley et al., 2018). A previous study evaluated the relationship between small vessel disease and aortic PWV in 167 hypertensive patients without a history of vascular or cerebrovascular disease (Henskens et al., 2008), demonstrating a positive correlation between PWV and white matter hyperintensities and the presence of silent lacunar infarcts. This correlation held even in younger patients (<50 years old) and was independent of vascular risk factors (i.e., MAP and age). Increasing the duration of the momentum of the pulse pressure increased the risk for tissue damage (Henskens et al., 2008).

A study that followed > 3000 patients evaluated the relationship between downstream small vessel damage and cognitive decline. Using forward compression wave intensity (FCWI), which measures the intensity of pulsatile waves traveling via the carotids toward the brain, it was concluded that elevated carotid artery FCWI was correlated with earlier cognitive decline, independently of other cardiovascular factors (Chiesa et al., 2019). In this study, participants were examined for changes in memory, executive function, and fluency over 13 years. Those with a higher FCWI in the carotid artery showed a 50% greater chance of developing cognitive dysfunction, even after adjusting for several factors, including education. In a cross-sectional study that included 668 females and males between 69 and 93 years of age, Mitchell et al. (2011) reported that higher carotid pulse pressure and carotid flow pulse amplitude were correlated with reduced cognitive function, assessed by tests of memory, executive function, and processing speed (Mitchell et al., 2011). An increase in PWV was shown to contribute to cognitive dysfunction through its negative impacts on white and gray matter volumes and its positive correlation with lacunar infarcts and white matter hyperintensities.

Increased vascular stiffness may result from increases in blood pressure variability (BPV) (Rothwell, 2010; Schillaci et al., 2012), defined as blood pressure fluctuations over time, where time can be measured in seconds to years (short-term, ≤ 24 h; mid-term, days/weeks; long-term, years) (Stevens et al., 2016, 2019). BPV is emerging as an independent predictor of cardiovascular risk and dementia (Merkler and Iadecola, 2017; Oishi et al., 2017). While the mechanisms underlying BPV are still unclear, augmented blood pressure fluctuations may be the result of reduced ability to buffer normal changes due to impaired vascular function (e.g., increased vascular stiffness), impaired baroreflex sensitivity and/or sympathetic nerve responses (Schillaci et al., 2012). BPV contributes to cardiovascular complications in hypertension, and antihypertensive agents that control it help prevent the development of cardiovascular events (Rothwell et al., 2010; Parati et al., 2012). A meta-analysis that included data from 27 articles and more than 12,000 individuals determined that greater short-term (24 h) BPV in humans was correlated with subcortical infarct, lacunae, white matter hyperintensities, cerebral microbleeds and/or enlarged perivascular spaces (Tully et al., 2020). Further supporting the adverse effect of increased BPV on neurovascular function, Oishi et al. (2017), provided evidence that BPV, is an independent risk factor in the development of AD and vascular cognitive impairment/dementia.

Multiple cellular and extracellular matrix mechanisms contribute to vascular stiffness, including elastin fragmentation, collagen deposition, advanced glycation endproduct (AGE)-mediated collagen and elastin cross-linking, impaired VSMC function and calcification, endothelial dysfunction, inflammation and oxidative stress, among others (Chirinos et al., 2019). While advances have been made in elucidating the impact of the renin-angiotensin system (RAS), especially Ang II (Forrester et al., 2018), on vascular fibrosis and arterial stiffness, the underlying cellular and molecular mechanisms remain elusive. Ang II in hypertension and arteriosclerosis is linked to inflammation and oxidative stress (Rodriguez-Iturbe et al., 2017). In VSMCs, elevated intracellular Ca^2+^, increased actin-myosin cross-bridge formation, cytoskeleton rearrangement, ROS, inflammation-related non-coding RNAs, as well as Ca^2+^-independent mechanisms involving RhoA-Rho kinase, protein kinase C and mitogen-activated protein kinase signaling lead to increased vasoconstriction and arterial remodeling, as recently reviewed by Touyz et al. (2018). Using a mouse model of arterial stiffness induced by administration of CaCl_2_ into the adventitia of the carotid artery, Sadekova et al. (2018) showed increased microglia and astrocyte reactivity in the hippocampus and frontal cortex. In this case, the observed gliosis was associated with NADPH-generated oxidative stress (Sadekova et al., 2018).

Taken together, while numerous human studies and observations found strong correlations between arterial stiffening and impaired cerebrovascular function in hypertension and cognitive decline, the apparent lack of direct mechanistic links and experimental evidence for well-defined molecular targets prevents therapeutic progress.

## Vascular Contributors to Cognitive Impairment and Dementia

The prevalence of hypertension, a modifiable risk factor for cardiovascular disease, increases with age. Importantly, in hypertensive patients, aging accelerates cognitive decline (Livingston et al., 2017). Thus, understanding the cellular mechanisms underlying hypertension- and aging-induced cognitive decline is pivotal for the development of therapeutic strategies in the prevention of vascular-related dementia.

The most common form of dementia is AD, a multifactorial neurodegenerative disease (Pomilio et al., 2020). Early presentations of AD include neurovascular dysfunction and microvasculature alterations (Sagare et al., 2013). Iturria-Medina et al. (2016) analyzed 7,700 brain images and dozens of plasma and cerebrospinal fluid biomarkers from the Alzheimer’s Disease Neuroimaging Initiative (ADNI), looking for abnormalities related to vascular, amyloid, metabolic, functional, and structural changes. They found that vascular dysfunction was an early pathological event. Importantly, cognitive decline was noticeable from the initial stages of late-onset AD and was associated with vascular alterations.

Mouse models of familial AD exhibit differences depending on the specific mutations and transgenes they carry. However, many present vascular alterations (Iadecola, 2017), including altered cerebral perfusion (Hebert et al., 2013; Guo et al., 2019), impaired NVC (Badhwar et al., 2013; Lu et al., 2019), capillary endothelial degeneration, thickening (Zlokovic, 2005), and loss of pericytes and BBB integrity (Sagare et al., 2013; Montagne et al., 2020), before behavioral changes and deposition of amyloid plaques. As in humans, these observations support the notion that vascular dysfunction is an essential factor in the onset of cognitive decline.

Increased blood pressure in mid-life is positively associated with cognitive decline or AD late in life (Skoog et al., 1996). A recent report of a clinical trial with a 6-year follow-up concluded that controlling blood pressure at a target level less than 140 mmHg decreased the incidence of mild cognitive impairment and lowered the risk of dementia (Sprint Mind Investigators for the Sprint Research Group, Williamson et al., 2019). Given the availability of potentially beneficial therapeutics and the lack of successful interventions for AD (Edwards Iii et al., 2019), antihypertensive drugs have been explored as AD treatments. A retrospective cohort analysis of patients taking antihypertensive RAS-acting medicines, including angiotensin-converting enzyme inhibitors (ACEIs) and Ang II receptor blockers (ARBs), reported that AD incidence was lower in women (both Caucasian and African American) and Caucasian men taking ARBs compared with their counterparts not taking RAS-acting drugs (Barthold et al., 2018). Furthermore, a meta-analysis found that controlling blood pressure with antihypertensive drugs in and of itself may have positive outcomes for cognitive function (Ding et al., 2020).

Targeting the RAS using Ang II receptor blockers has proven beneficial for cognition in both human patients and animal models. AD mice (*J20* model) treated with losartan showed improved posterior cerebral artery reactivity and restoration of functional hyperemia, this despite the fact that amyloid deposits in the brain did not decrease, and even showed a tendency to increase. Remarkably, losartan improved outcomes in cognitive tests in AD mice. At the cellular level, losartan decreased the oxidative state/stress and astrogliosis (Royea et al., 2017). These results are in agreement with those of another study in which losartan treatment improved cerebrovascular and cognitive function in older mice (12 months) (Ongali et al., 2014). These observations argue against a link between Ang II receptors and amyloid pathology-related cognitive dysfunction, but favor the idea that improving vascular function with the use of antihypertensive drugs could positively impact cognitive outcomes in AD. In humans, telmisartan treatment was associated with increased resting CBF and improved cognitive function (Kume et al., 2012), supporting the use of antihypertensive treatments to improve vascular function and cerebral perfusion. In a randomized controlled trial, 6-month treatment with telmisartan, an AT_1_R blocker, in probable AD patients with essential hypertension decreased proinflammatory cytokines in the CSF and increased Aβ_1__–__42_, indicative of augmented clearance (Li et al., 2012). An important point to consider, however, is that not all drugs that block Ang II receptors are helpful in restoring cognitive function (Trigiani et al., 2018) or are effective in long-established AD pathology, as seen in mice (Ongali et al., 2016).

The use of antihypertensive drugs has also been proposed as a strategy for ameliorating amyloid pathology. A recent study in which regulation of the RAS was enhanced through ACE2 in the Tg2676 mice model of AD showed that spatial memory was improved in association with a reduction in soluble and insoluble Aβ in the hippocampus (Evans et al., 2020). Here, however, vascular function was not evaluated. Ang-II–infused hypertensive mice exhibit increased production of amyloid precursor protein (APP) binding proteins (Csiszar et al., 2013). Yet, no changes in the production of APP-processing enzymes that participate in the amyloidogenic process were found.

Further studies are needed to determine the effects of antihypertensive drugs and their mechanisms of action concerning the vascular component of AD. These drugs might be useful in the prevention of AD and dementia by preserving vascular function. However, their potential to mitigate the contribution of amyloid pathology cannot be ruled out. In this latter context, it could be that the driver of amyloid pathology in hypertensive models and patients relates to the deleterious effects of hypoxia/ischemia on vascular function, as these conditions activate the processing of APP (Shiota et al., 2013).

Whether independent or as a result of hypertension, evidence also supports a link between arterial stiffness and AD pathology and related dementias. Notably, arterial stiffness is a contributing factor to pulsatile hemodynamics, cerebral hypoperfusion, and microvascular damage. In older adults, arterial stiffness is positively correlated with an increased incidence of cerebral small vessel disease (Hughes et al., 2015; Chirinos et al., 2019). Rivera-Rivera et al. (2020) showed that cerebrovascular stiffness was increased in middle-aged AD patients. In this work, the authors employed high temporal resolution 4D flow magnetic resonance imaging (MRI) to assess transcranial PWV in 160 participants classified according to cognitive function. They found that transcranial PWV was significantly increased in subjects with myocardial infarct or AD.

Notably, maladapted cerebral hemodynamics could lead to alterations in the structural and functional connectivity of the brain, as exemplified by white matter lesions/hyperintensities, a symptom of small vessel disease and parenchymal artery dysfunction (Bagi et al., 2018). At rest, the brain exhibits slow blood flow fluctuations that are interpreted as providing effective connectivity between regions comprising what is known as the default mode network (Raichle, 2015). Reduced functional connectivity between the hippocampus and other brain regions is found in AD patients (Allen et al., 2007), and has been shown to correlate with behavioral changes (Multani et al., 2019).

As is the case in hypertension and aging, diminished cerebral perfusion, perhaps initiated by morphological alterations of the microvasculature, may play a role in the development of AD through increases in oxidative stress and compromised neurovascular interactions. Decreased oxygen delivery to brain regions, including the hippocampus, leads to neurodegeneration and cognitive decline (de la Torre, 2000; Mehla et al., 2018). Tong et al. (2012) showed therapeutic benefits from simvastatin treatment in a mouse model of AD. Simvastatin lowered ^O_2^–^ production and normalized antioxidant superoxide dismutase 2 levels. Importantly, simvastatin restored cerebrovascular function and NVC in both aged (12 months) and adult mice (6 months). Short- and long-term memory was restored in aged adults but not aged AD mice, even without effects on plaque or amyloid β (Aβ) levels.

At the level of the NVU, Aβ has been shown to cause pericyte-induced capillary constriction in both AD patients and an AD mouse model. The underlying mechanism was found to involve the generation of ROS (mainly via NOX4), resulting in the release of the potent vasoconstrictor agent, endothelin-1 (ET-1). ET-1, in turn, acted through ET_*A*_ receptors to evoke pericyte constriction. Notably, Aβ-evoked constrictions were prevented by blocking NOX4 or ET_*A*_ (Nortley et al., 2019). A recent study showed that secretion of APOE4 by pericytes resulted in activation of cyclophilin A and stimulation of downstream protein matrix metalloproteinase-9. Increased inflammation in pericytes, and possibly endothelial cells, increased BBB leakiness in the hippocampus and parahippocampus brain areas associated with learning and memory.

Aβ peptide aggregation in the wall of cerebral microvessels (cerebral amyloid angiopathy) also impairs vascular function (Kimbrough et al., 2015) and is a risk factor for vascular cognitive impairment. Using a combination of *ex vivo* and *in vivo* imaging approaches in mice, Kimbrough et al. (2015) found that astrocyte endfeet were separated from cerebral vessel segments that showed amyloid deposits and that astrocyte stimulation resulted in poor vascular responses. In contrast, astrocyte-induced vascular responses were intact in vessel segments that lacked amyloid deposits (Kimbrough et al., 2015). Collectively, these observations support a role for astrocytes in the regulation of CBF in AD.

The importance of vascular homeostasis in AD and associated dementias has become an important shift in the quest to identify the factors contributing to cognitive decline (Csiszar et al., 2017). Collectively, the above findings call for controlled clinical trials and advanced animal models to help disentangle mechanisms targeted by antihypertensive and antioxidant treatments in the therapeutic benefits of these drugs in the setting of vascular-related cognitive impairment and dementia.

## Conclusion

**FIGURE 1:**
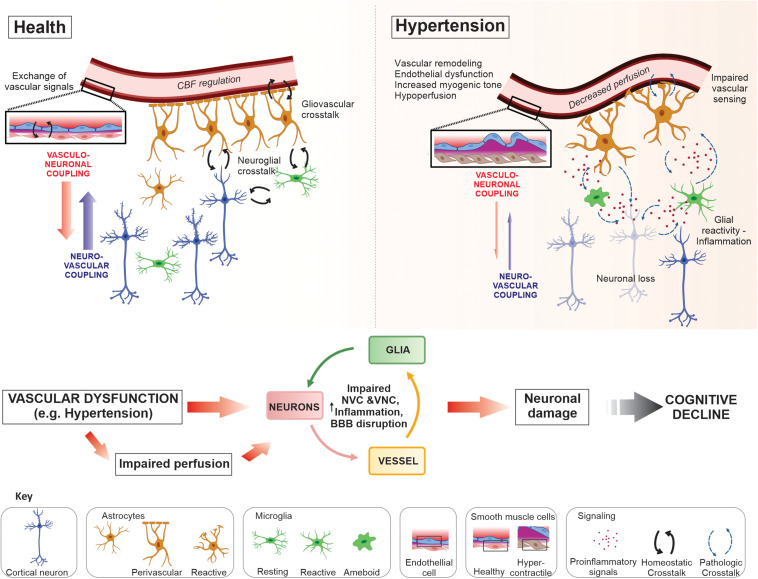
Neurovascular unit crosstalk interactions in health and disease. In the healthy brain dynamic crosstalk interactions at the neurovascular unit (NVU), via multiple signaling modalities, maintain a homeostatic environment optimal for neuronal function. Astrocytes and microglia are target of neuronal signals, i.e., through specific receptors or extracellular vesicles, to which they respond exerting several functions in coordination (for example, neuronal support, synaptic development and formation, phagocytosis and modulation of neuronal activity). The NVU crosstalk also includes the release of vasoactive signals from vascular, glial and neuronal cells. Together, signals from these cells aid in the regulation of cerebral blood flow (CBF), a process requiring both neurovascular (NVC) and vasculo-neuronal coupling (VNC); maintenance of the blood–brain barrier (BBB); immune surveillance; ionic and neurotransmitter homeostasis; metabolic support; and neurotransmission, to name a few. Disease conditions such as hypertension evoked changes at the NVU that progressively impaired communication between cells shifting the normal crosstalk into a pathological state. Vascular dysfunction, including remodeling, endothelial and vascular smooth muscle cell (VSMC) dysfunction, vascular and glial inflammation (i.e., astrogliosis and microgliosis), all contribute to impaired perfusion to the NVU and loss of BBB integrity. The constitutive presence of these deleterious processes diminishes the stability of the NVU and impair neuronal function contributing to neurodegeneration and, ultimately, cognitive decline.

This review highlights key crosstalk dynamics at the NVU, which maintain homeostasis in the brain (Figure 1). We discussed key molecular regulators of parenchymal arteriole vascular tone and the pathological changes these arterioles undergo in disease conditions, primarily hypertension. In addition, we describe how other components of the NVU (e.g., glial cells) may contribute to disease progression (Figure 1). Clearly, more work is needed to understand the cellular mechanisms underlying loss of function at the NVU and how vascular alterations (i.e., vascular stiffness, remodeling, and astrocyte-vascular uncoupling) impact homeostatic as well as energy-dependent processes in the brain. While vascular dysfunction is strongly linked to cognitive decline, many factors such as the cellular targets involved in disease-specific insults (i.e., CBF, intravascular pressure, ischemia, inflammation) as well as the consequences these have on cerebral perfusion and neuronal functional remain ill define. Thus, future studies addressing the cellular and molecular players in the loss of physiological crosstalk at the NVU are needed to shed light on early processes, with therapeutic potential, to prevent what in later life manifests as vascular cognitive impairment and dementia.

## Author Contributions

All authors listed have made a substantial, direct and intellectual contribution to the work, and approved it for publication.

## Conflict of Interest

The authors declare that the research was conducted in the absence of any commercial or financial relationships that could be construed as a potential conflict of interest.
